# Molybdenum-, Vanadium-, and Tungsten-Containing Materials for Catalytic Applications

**DOI:** 10.3390/ma15051720

**Published:** 2022-02-25

**Authors:** Dominique Agustin, Jana Pisk

**Affiliations:** 1 LCC-CNRS (Laboratoire de Chimie de Coordination), 205 Route de Narbonne, BP44099, CEDEX 4, 31077 Toulouse, France; 2 Department of Chemistry, IUT Paul Sabatier, Université Paul Sabatier, University of Toulouse, Av. G. Pompidou, CS20258, 81104 Castres, France; 3 Department of Chemistry, Faculty of Science, University of Zagreb, Horvatovac 102a, 10000 Zagreb, Croatia

As chemists, we are still fascinated by the magic of nature. Indeed, natural metalloenzymes can achieve a high number of chemical transformations without the action of humans [1]. From the chemist’s point of view, the nature of coordination species and the environment around metals determine their activity [2]. Metalloenzymes are active in several reactions [3], particularly in oxidation processes with the assistance of molybdenum- [4], vanadium- [5], and tungsten-based compounds [6]. The activity of those species is quite large and the advantage lies in the stability of such complexes in aqueous media or under air [7]. According to the reported academic research known for decades, other metallic species are investigated to be active and are mainly composed of an inorganic part, such as as polyoxometalates (POMs) [8,9,10,11,12,13]. Among the several applications of POMs within several domains [8,9] and focusing on catalytic applications [10,11], POMs are considered as molecular models of metal oxides by mimicking the surface of metal oxides [12] present in nature (as rocks) and acting within some catalytic processes (mainly heterogeneous ones) [11].

Since our research domain is the catalytic applications of oxo-molybdenum and oxo-vanadium coordination complexes [14,15,16,17,18] and POMs [19,20,21], such as molecular [19,20] or supported catalysts [21], with emphasis on biomass-based substrate valorisation [22,23,24], we proposed to edit this Special Issue with the aim of collecting research of other international groups within the domain to show the diversity of possible catalyzed reactions using metal complexes. High catalytic activity with relatively low metal loading is an asset, from relatively low cost to more sustainable processes. Catalytic processes containing those elements are of a growing interest, notably heterogeneous ones, in terms of reuse and recycling.

The Special Issue highlights some recent advances in the development of Mo-, V-, and W-containing catalysts, including polyoxoanions and bulk materials (e.g., mesoporous materials, surfaces, etc.), with the involvement of those elements in catalytic materials. The emphasis is on recent trends, including materials processing (preparation and characterization) with their catalytic applications from simple reactions with model substrates to more complex and challenging ones.

The four original research papers collected in this thematic issue cover different aspects, dealing with processes using POMs non-supported and silica-supported (porous and non-porous) materials, managing different catalytic reactions, and exhibiting different approaches.

The article named “Vanadium-Substituted Phosphomolybdic Acids for the Aerobic Cleavage of Lignin Models—Mechanistic Aspect and Extension to Lignin” is a research article from Paris (France) [25]. Al-Hussaini, Launay, and Galvez developed a very interesting study based on lignin cleavage. Lignin is a polymer with several types of linkages between phenolic species. The authors used the ability of the heterometallic polyacid of general formula H_6_[PMo_9_V_3_O_40_]. This compound was synthesized using a variant of a known method under hydrothermal conditions. This POM was tested for the aerobic cleavage of two lignin models bearing a *β*-O-4 bond in lignin. The article points out the effect of several parameters for the reactions and application to a real case, using an organosolv wheat straw lignin. From the obtained results, a mechanism of cleavage was proposed.

Wang, Wang, Shang, Ren, Yue, and He from Fudan (China) presented, in the article titled “H_3_PMo_12_O_40_ Immobilized on Amine Functionalized SBA-15 as a Catalyst for Aldose Epimerization” [26], the use of a heterogeneous catalytic system based on mesoporous silica (SBA-15) purposely functionalized with APTES for the immobilization of H_3_PMo_12_O_40_. Several POMs loadings were investigated, and catalytic objects were used to epimerize glucose in water, aiming to valorize cheap sugar into high value ones. The aim was to transform glucose into mannose, and the results showed good selectivity with better activation energy (a gain of 16 kJ mol^−1^) with immobilized catalyst vs. the molecular version. Other aldoses (among them mannose, arabinose, and xylose) were tested.

Another type of supported catalysts was presented in “Organic Solvent-Free Olefins and Alcohols (ep)oxidation Using Recoverable Catalysts Based on [PM_12_O_40_]^3−^ (M = Mo or W) Ionically Grafted on Amino Functionalized Silica Nanobeads” [27]. Wang, Gayet, Guillo, and Agustin from LCC-CNRS Toulouse and Castres (France) showed how H_3_PMo_12_O_40_ and H_3_PW_12_O_40_ could be immobilized on non-porous silica nanoparticles functionalized with APTES. The species were fully characterized by using different methods more specifically to quantify APTES and POM loading on the surface. The objects were tested for epoxidation of classical cyclic olefins, one terpene (limonene), and the oxidation of cyclohexanol. The catalysts could be recycled several times.

The last article of this Special Issue is a collaboration between Erlangen (Germany) and DTU at Lyngby (Denmark). “Ru-Doped Wells–Dawson Polyoxometalate as Efficient Catalyst for Glycerol Hydrogenolysis to Propanediols” written by Modvig, Kumpidet, Riisager, and Albert exhibits the valorization of glycerol in aqueous media using a Ru-doped Wells–Dawson polyoxometalate of general formula α2-K_x_P_2_MW_17_O_61_ (M = Ru, Pd, Pt). [28] Those species have been used to perform hydrogenolysis of glycerol into propanediols. The POM containing Ru was more active than Pd and Pt. In addition to catalysts loading, other parameters that influenced reactivity included H_2_ pressure, stirring rate, etc.

In conclusion, the four articles published in this Special Issue show the richness of polyoxometalates as (supported/unsupported) catalysts for different reactions working in aqueous media or without organic solvent. The results presented exhibit important application, from the biomass valorization, downstream byproducts of industrial processes towards new materials, or cleaner processes. This is of crucial importance for a sustainable future in today’s societies. We hope that this thematic issue can stimulate further research in the field.
